# Mismatch negativity as EEG biomarker supporting CNS drug development: a transnosographic and translational study

**DOI:** 10.1038/s41398-021-01371-1

**Published:** 2021-04-29

**Authors:** Simon Loiodice, Wilhelmus H. Drinkenburg, Abdallah Ahnaou, Andrew McCarthy, Geoffrey Viardot, Emilie Cayre, Bertrand Rion, Valérie Bertaina-Anglade, Marsel Mano, Philippe L’Hostis, Christophe Drieu La Rochelle, Martien J. Kas, Philippe Danjou

**Affiliations:** 1 Biotrial Pharmacology, 7-9 rue Jean-Louis Bertrand, 35042 Rennes, France; 2 Department of Neuroscience Discovery, Janssen Research & Development, a Division of Janssen Pharmaceutical NV, Turnhoutseweg 30, B-2340, Beerse, Belgium; 3 Groningen Institute for Evolutionary Life Sciences, University of Groningen, P.O. Box 11103, 9700 CC, Groningen, The Netherlands; 4 Lilly Research Laboratories, Windlesham, Surrey, GU20 6PH UK; 5 Biotrial Neuroscience, Avenue de Bruxelles, 68350 Didenheim, France

**Keywords:** Neuroscience, Biomarkers

## Abstract

The lack of translation from basic research into new medicines is a major challenge in CNS drug development. The need to use novel approaches relying on (i) patient clustering based on neurobiology irrespective to symptomatology and (ii) quantitative biomarkers focusing on evolutionarily preserved neurobiological systems allowing back-translation from clinical to nonclinical research has been highlighted. Here we sought to evaluate the mismatch negativity (MMN) response in schizophrenic (SZ) patients, Alzheimer’s disease (AD) patients, and age-matched healthy controls. To evaluate back-translation of the MMN response, we developed EEG-based procedures allowing the measurement of MMN-like responses in a rat model of schizophrenia and a mouse model of AD. Our results indicate a significant MMN attenuation in SZ but not in AD patients. Consistently with the clinical findings, we observed a significant attenuation of deviance detection (~104.7%) in rats subchronically exposed to phencyclidine, while no change was observed in APP/PS1 transgenic mice when compared to wild type. This study provides new insight into the cross-disease evaluation of the MMN response. Our findings suggest further investigations to support the identification of neurobehavioral subtypes that may help patients clustering for precision medicine intervention. Furthermore, we provide evidence that MMN could be used as a quantitative/objective efficacy biomarker during both preclinical and clinical stages of SZ drug development.

## Introduction

Central nervous system (CNS) drug discovery is facing a high attrition rate in clinical trials with only a small number of innovative drugs reaching the market^1–3^. A lack of translation of therapeutic efficacy from basic research into new medicines is particularly important for neuropsychiatric diseases^4–6^ where the need to develop more predictive animal models as well as quantitative biomarkers has been pointed out^1,7–9^.

Currently, neuropsychiatric disorders are diagnosed and clustered, based on their symptomatology but not on their underlying etiology^8^. While this approach dominated basic clinical management over the last decades, it often lacked underpinning by the underlying neurobiology associated with individual symptoms and hence did not optimally foster the discovery and development of neurobiology-based novel treatments. Also the concept may apply to individualized medicine, helping to select the best treatment on the basis of a biology-driven marker, similarly to antibiogram for infectious diseases. The idea that clustering patients based on neurobiology, irrespective to the symptoms, could improve the discovery/development of better treatments for patients, has raised^8,10,11^ and constitutes the mission of the Innovative Medicines Initiative (IMI) PRISM consortium^8^. The goal of such transnosographic approach is to rely on quantitative biological parameters and evolutionarily preserved neurobiological/neuropharmacological systems to allow back-translation from clinical to nonclinical research^11^.

In this respect, electroencephalography (EEG)-mediated measurement of neuronal activity in a variety of modalities is attractive since this technique can be used in a comparable manner in man and most animals^8^. Accumulating data have drawn attention on two particular EEG-detectable phenomena based on event-related potentials (ERP), namely mismatch negativity (MMN) and auditory steady-state response (ASSR), which were both shown to be impaired in a number of neuropsychiatric conditions, including schizophrenia (SZ) and Alzheimer’s disease (AD) (see Danjou et al.^12^ for review): two disorders for which information processing and cortical pathways are also known to be affected.

MMN is an ERP complex generated in response to unattended changes within a stimulation sequence. It is passively evoked when a sequence of repetitive standard stimuli is occasionally interrupted by infrequent/deviant stimuli that differ in some physical dimension (pitch, duration) and it is thought to reflect an automatic or preconscious process of detecting a “mismatch” between a deviant stimulus and a sensory-memory trace, i.e., via deviance detection^13,14^. Auditory MMN is often used to study working memory as it is an informative probe of the neural substrates of sensory processing abnormalities and relies on a distributed network of frontotemporal cortical sources underlying passive auditory sensory discrimination^13,15^.

In line with the IMI PRISM working hypothesis, we aimed to investigate whether, similarly to symptoms such as cognitive dysfunction, electrophysiological impairments may overlap within SZ and AD patients using MMN. We also sought to back-translate this approach into animal models in order to assess the value of MMN-like as EEG biomarker in early drug development.

## Materials and methods

### MMN in schizophrenic/Alzheimer’s patients

#### Participants

Eligible patient and healthy control participants were identified via patient cohort registers and clinical programs affiliated with the participating centers. The study has been conducted at three research sites in The Netherlands (University Medical Center Utrecht, VU University Medical Center Amsterdam, and Leiden University Medical Center), and two research sites in Spain (both located in Madrid: Hospital General Universitario Gregorio Marañón and Hospital Universitario de La Princesa). In light of data suggesting that impairment of MMN response, as well as social withdrawal, could be observed both in SZ and AD patients, it was hypothesized that MMN response may correlate with the level of social withdrawal, regardless of the diagnosed disease^8,12,16^. Therefore, patient participants were selected to differ by level of social withdrawal (high vs. low), according to their researcher-rated score on a subset of items from the WHODAS-2 (social withdrawal) scale. Low social withdrawal was defined as a score ≤10, whereas high social withdrawal was denoted by a score ≥11.

Key exclusion criteria for patients included social withdrawal due to external circumstances or disease-unrelated disabilities (e.g., lack of access to transport, lack of mobility, and facial disfigurement), whereas right-handedness/ambidextrousness was an inclusion criterion. Alzheimer’s disease patients were additionally required to fall between the ages of 50 and 80 (inclusive), meet the National Institute on Aging and the Alzheimer’s Association (NIA-AA) criteria for probable AD, and have an MMSE score of 20–26 (inclusive). SZ patients were required to fall between the ages of 18 and 45 (inclusive), meet the DSM-IV diagnosis of SZ with at least one confirmed psychotic episode but a maximum of 10-year disease duration since diagnosis, and be on stable doses of medication for at least 8 weeks prior to screening. Patient participants were excluded if they presented very severe disease symptoms (e.g., a score of ≥22 on the 7-item PANSS-positive symptom factor for SZ, a score <20 on the MMSE for Alzheimer’s Disease), or had a current DSM-IV diagnosis of major depressive disorder (as assessed by the MINI or scored ≥16 on the QIDS-SR16), or suffered from drug or alcohol dependence within the three years prior to screening, or had any contraindications for MRI studies.

Healthy control participants were required to fall into the same two age brackets as the patient groups and to be right-handed or ambidextrous, and were excluded if they had a past or current diagnosis of an Axis-I psychiatric disorder as determined by the MINI, mild depressive symptoms as indicated by a score of 5 on the QIDS-SR16, or any contraindications for MRI studies. For complete inclusion/exclusion criteria, see Bilderbeck et al.^17^.

The final sample for the PRISM clinical study consisted of 165 participants: 52 AD patients, 56 SZ patients, 29 younger, and 28 elderly healthy controls. The characteristics of the sample in terms of social withdrawal levels, gender, ethnicity, age, and years in education can be visualized in Table 1.

**Table 1:** Characteristics of the IMI PRISM clinical study sample.

	AD = 52	SZ = 56	Young HC = 29	Old HC = 28
N low SW (patients only)	31	27		
N high SW (patients only)	21	29		
N male	29	40	17	15
N female	23	16	12	13
N White	51	41	28	28
Age	68.75, SE = 1.00	30.77, SE = 0.85	28.72, SE = 1.37	67.07, SE = 1.33
Years in education	15.10, SE = 0.77	14.96, SE = 0.51	17.17, SE = 0.48	16.71, SE = 0.92

*N* Number of, *AD* Alzheimer’s disease, *SZ* schizophrenia, *HC* healthy controls, *SE* standard error of the mean, *SW* social withdrawal.

#### Auditory mismatch negativity (MMN) procedure

In this study, we applied the methodology used in PRISM for MMN induction, recording, and analyses. These adhere to the guidelines derived from Duncan and colleagues^18^ and were previously described^12^. To assess MMN, subjects received auditory stimulation (binaural). The stimulus consisted of three types of auditory stimuli (a standard and two deviant tones). One deviant tone differs from the standard tones (1000 Hz, 50 ms) in duration (1000 Hz, 100 ms). The second type of deviant differs from the standard tone in frequency (1050 Hz, 50 ms).

**Fig. 1:**
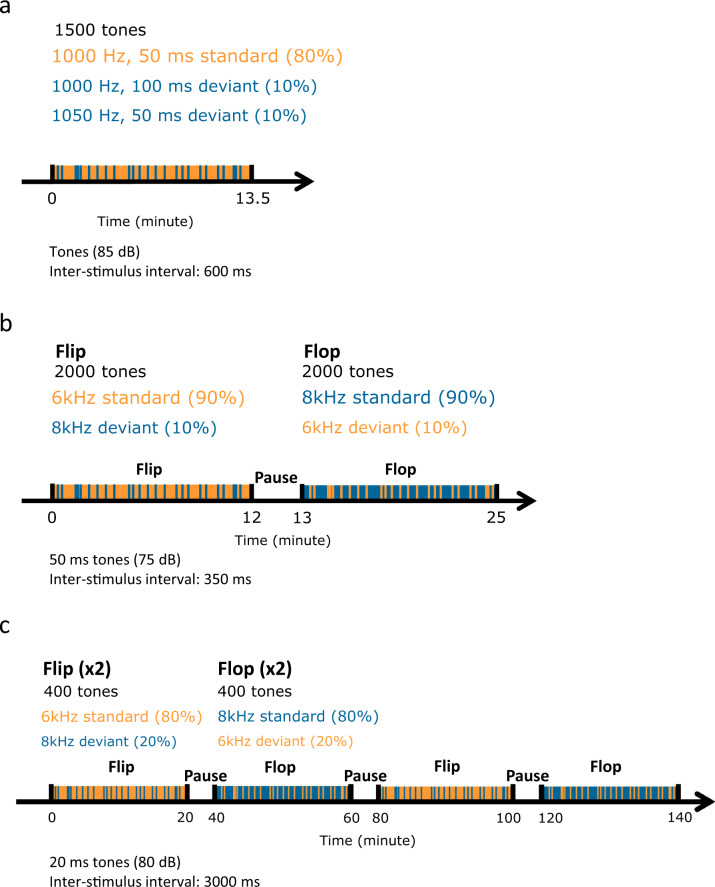
Auditory MMN procedure across species. Schematic overview of the auditory stimulation protocols used to elicit MMN response in human subjects (**a**), rats (**b**), and mice (**c**).

The total duration of MMN recording was 13.5 min and corresponds to a total of 900 s and corresponds to ~1500-tone presentation (see Fig. 1a). Among them, 10% were duration-deviant tones and 10% were frequency-deviant tones. The sound level was 85 dB (for all types of stimuli), with 5-ms rise and fall times, and with tones played using headphones. The interstimulus interval was 600 ms. The intensities of the sounds were similar to the ones used mostly in ERP studies in schizophrenia^15,19,20^. Triggers, synchronized with tone emission, were recorded on an ad hoc channel of the EEG acquisition system. The time when trigger occurs was considered as time 0 ms of the ERP to assess. No additional control sequence was applied to minimize patient discomfort considering the overall number of procedures^12^ although this could be seen as a limitation.

#### EEG recording and processing

EEG signals were collected using EEG caps with the position of the 64 EEG electrodes following the international 10–20 Jasper system (eego system from ANT; pass band: 0.1–70 Hz; sampling rate: 2048 Hz). Impedances of all electrodes were kept lower than 20 kΩ. In order to improve artifact rejection, two electro-oculographic (EOG) channels were simultaneously recorded using silver cup electrodes to monitor ocular movements and eye blinks. In order to reduce electromagnetic noise due to the main power supply, a digital narrow 50-Hz-centered notch filter was applied to data. During data acquisition, subjects were seated on an armchair bed in a quiet environment and watched a short silent video (in order to provide a visual distraction). None of the participants exhibited hearing problems as checked by a simplified tonal audiometric evaluation performed on study subjects at screening.

EEG data were first preprocessed by performing filtering, epoching (i.e., [−200 + 500] ms around the stimuli), and various steps of automatic artifact reduction and rejection^21–24^. Artifact rejection and ERP constructions were carried out using Matlab. Next, the clean EEG was used to extract biomarkers^25^.

Epochs were then grouped based on their corresponding stimuli type (i.e., standard or deviant).

For each electrode, the MMN ERPs were obtained by subtraction of the average ERP of standard stimuli from the average ERP of deviant stimuli, while the parameters extracted from the MMN difference wave were

- *Peak amplitude* (µV): amplitude of the negative peak in time window [90, 290] ms after the stimulus.
- *Peak latency* (ms): latency of the above peak in time window [90, 290] ms after the stimulus.
- *Area under the curve or AUC* (µV.ms): integral of the curve (signed area) on the [90, 290 ms] window.
- *Average amplitude* (µV): average amplitude was calculated on each electrode using a 100-ms window around the latency of the negative peak detected from the average MMN ERP of six electrodes located above the frontal lobe [“Fz”, “FCz”, “F1”, “F2”, “FC1”, “FC2”].

### Mismatch negativity in PCP-modeled rats

#### Animals

Adult male Sprague-Dawley rats (200–230 g) from Janvier Labs (Saint Berthevin, France) were maintained in a controlled environment (lights on 08:00–20:00, ±22 °C) with food and water freely available. They were single-housed after the EEG surgery. This study was carried out in AAALAC-accredited facilities in strict accordance with the European Communities Council Directive (2010/63/EU) guidelines for the care of laboratory animals. The protocol was approved by the Biotrial Pharmacology Committee on the Ethics of Animal Experiments “Comité de Réflexion Ethique en Expérimentation Animale” (CR2EA), and in accordance with French Research Ministry regulations. All possible efforts were made to minimize discomfort and ensure the well-being of the animals.

#### Surgical procedure

A cohort of 26 rats was used for stereotaxic implantation of EEG leads and telemetry device. Anesthesia was induced and maintained with isoflurane, while animals were placed in a stereotaxic frame (David Kopf Instruments, CA, USA). A bipotential implant (TL11M2-F40-EET, Data Sciences International, St Paul, MN, USA) was used for radiotelemetry recording of electrophysiological signals in freely moving rats. The four leads of the sensor were fixed on the skull with screws and cement at the following stereotaxic coordinates^26^: AP: + 2.0 mm from bregma, ML: −1.5 mm (active electrode) with a reference on the left cerebellum, i.e., AP: −11.0 mm from bregma, ML: −3.0 mm (Channel #1), and AP: −4.8 mm from bregma, ML: + 7 mm (active electrode) with a reference on the right cerebellum, i.e., AP: −11.0 mm from bregma, ML: + 3.0 mm (Channel #2). These stereotaxic coordinates were slightly adapted from previous studies^27,28^ and selected based on preliminary experiments performed in the lab.

The telemetry transmitter itself was placed subcutaneously along the animal’s flank and attached with nonabsorbable sutures. The skin incisions were then closed with a resorbable suture.

Postsurgical analgesia was ensured by subcutaneous administration of buprenorphine (Buprecare^®^, 0.03 mg/kg, bid) and meloxicam (Metacam^®^, 2 mg/kg). At least 10 days were allowed to ensure a complete recovery from the surgery. During this recovery period, the animals were weighed and observed daily and the sutures were examined and disinfected when needed with antiseptic solution (Vetedine^®^).

Three rats exhibited clear signs of implant rejection (open wound) during the postoperative recovery period and were sacrificed for human reason before baseline MMN acquisitions. Ten and thirteen rats were allocated to the vehicle and PCP group, respectively.

#### Drugs

Phencyclidine (PCP) (Sigma, France) was freshly dissolved each day of dosing in saline.

Saline or PCP (5 mg/kg, ip) was subchronically administered twice daily (10 am and 5 pm) from day 1 to day 7 as previously described^29,30^. Next, animals were tested after a wash-out period of 7 days after the last administration of either saline or PCP. During this period, animals were left in their home cage without any treatment.

#### Auditory mismatch negativity (Flip-Flop) procedure

All of the electrophysiological recordings were performed 7 days after the last day of PCP administration and took place between 9 am and 1.30 pm in the home-cage environment.

The telemetry data were collected using an acquisition system from Data Sciences International (St Paul, MN, USA). During the experimental session, the animals were individually housed in cages placed on the receiver panel. EEG signal was monitored and stored on the hard drive of a personal computer using HEM software (version 4.3, Notocord France).

The stimulation protocol used is illustrated in Fig. 1b and has been adapted from the protocol used in a clinical trial by BIOTRIAL described above, from previously published work^27^ and from preliminary experiments performed in the lab. Briefly, EEG was continuously recorded during a 25-min session of acoustic stimulation using an oddball flip-flop paradigm. During the first 12 min (flip), animals were exposed to approximately 2000 tones with 90% of sounds at 6 kHz (standard sound) and 10% at 8 kHz (deviant sound) with a fixed interstimuli interval of 350 ms. After a 1-min pause (silent), another 12 min of acoustic stimulation was performed (flop) with approximately 2000 stimuli with 90% of sounds at 8 kHz (standard sound) and 10% at 6 kHz (deviant sound) and a fixed interstimuli interval of 350 ms. Auditory stimuli were delivered by sound card controlled by ad hoc scripts running under E-Prime v2 software (Psychology Software Tools, Sharpsburg, PA, USA). The same script sends synchronization triggers to the acquisition system in order to allow EEG epochs averaging to obtain ERPs. Sound stimuli were delivered through a speaker mounted above the experimental cage. Each stimulus was calibrated as follows: using a sound calibrator (Laserliner SoundTest Master, Germany) prior to the test: 75 dB, 50 ms (fade-in: 5 ms, fade-out: 5 ms).

Signal processing was performed using ad hoc Matlab scripts. Continuous EEG was split into epochs surrounding each auditory stimulus. EEG epochs start 50 ms prior to stimulus onset and last 350 ms. Low-pass filter was applied on each epoch. This filter is a zero-phase-shift Butterworth filter admitting a 50-Hz cut-off frequency and a 22-order to remove high-frequency components that are noisy or irrelevant for the current study. The average value prior stimulus onset was subtracted from the whole epoch to achieve a baseline correction. Automatic epoch rejection relies on the threshold method: if the amplitude of any sample of an epoch exceeded 300 µV, this epoch was rejected from the analysis. Linear detrending was performed on each remaining epoch. Epoch set was slipped into four subsets matching with the two types of stimulus and the probability of occurrence (4 classes: 6 Hz or 8 Hz during flip or flop). Averaging all remaining epochs within each subset was performed to obtain a single ERP for each class and each recording. The difference between ERP obtained from deviant stimuli minus standard stimuli leads to new ERPs where MMN can be measured: the MMN was estimated by calculating the integral on the [65–105]-ms interval. Parameters were extracted from those individual ERPs. Grand average ERPs are the mean of individual ERPs for each condition.

To counterbalance the individual variability previously observed in our setting (preliminary experiments, data not shown), the quantitative comparison between vehicle- and PCP-treated rats was made after baseline normalization of the MMN area (i.e., individual MMN area post treatment against MMN area prior treatment in the same individual).

#### Behavioral procedures

A cohort of 27 rats (*n* = 13 vehicle and *n* = 14 PCP) was used for behavioral testing. All of the behavioral procedures were initiated 14 days after the first PCP administration and took place between 9.30am and 12.30 pm.

Spontaneous locomotor activity was measured using an automated actimetry system. Animals were individually placed into cages (33 × 21 × 18 cm) positioned in an activity meter (Imetronic system, France) allowing automatic recording of horizontal animal displacements through infrared beams for a period of 120 min (number of infrared beams break).

Memory performance was assessed using a novel object-recognition (NOR) test. The experimental arena consists of a square wooden box (60 × 60 × 40 cm) painted dark blue with a Plexiglas^®^ floor. The arena was placed in a dark room illuminated only by halogen lamps, oriented toward the ceiling and giving a uniform, dim light in the box (around 60 lux).

For each NOR experiment, the rats were subjected to two trials separated by an intertrial interval of 120 min. During the first trial (acquisition trial, T1), the rats were placed in the arena containing two identical objects, and the time required by each animal to complete 15 s of object exploration was determined, with a cut-off time of 5 min. Exploration was considered to be pointing the nose at a distance of <2 cm from the object and/or touching the object and was recorded with a timer in real time via observation by a trained experimenter, who was unaware of the treatment conditions. For the second trial (testing trial, T2), one of the objects presented in the first trial was replaced with an unknown object (novel object), the rats were placed back in the arena for 3 min, and the time spent in active exploration of the familiar (tF) and novel (tN) objects was recorded.

The difference between the time spent in exploration of the novel object and of the familiar object (Delta = tN–tF) was calculated.

### Mismatch negativity in APP/PS1 and WT mice

#### Animals

Experiments were conducted in adult APP/PS1-21 and wild-type (BL/6J) mice of 13-month age: the APP/PS1-21 transgenic mouse model for AD coexpresses KM670/671NL-mutated amyloid precursor protein (APP) and L166P-mutated presenilin 1 (PS1) under the control of a neuron-specific Thy1 promoter element: the most aggressive familial AD mutation identified so far is the Leu-to-Pro mutation at position 166 of presenilin 1 (PS1). Mice were bred and raised at the Janssen Transgenic Facility (Beerse, Belgium). All experiments were conducted in strict accordance with the guidelines of the Association for Assessment and Accreditation of Laboratory Animal Care International (AAALAC) and with the European Communities Council Directive of September 22, 2010 (2010/63/UE), with approval from the local ethical committee. Ten animals of each genotype were housed in individually ventilated cages located in a sound-attenuated chamber maintained under controlled environmental conditions (12-h L–D cycle with lights on at 7 pm) with food and water available ad libitum.

#### Surgical procedure

Surgery was performed under general isoflurane anesthesia. Epidural recording electrode screws were stereotactically inserted onto the frontal cortex (AP: + 1.95 mm from bregma and ML: + /− 1.5 mm), reference electrode (AP: −0.5, ML −0.5), and the ground electrode placed on the cerebellum^31^. Electrodes were connected as above into a 10-hole connector and fixed with dental cement to the skull. During surgery, a subcutaneous injection of the analgesic Piritramide (Dipidolor^®^, 0.025 mg/kg) was administered to mice. As a further precaution to minimize possible perisurgical pain, a local spray analgesic (Xylocaine, 10%) was applied at the surgery site. After surgery, animals were singly housed and were allowed to recover for at least 10 days. During this recovery period, the animals were daily weighed and monitored for health.

#### Auditory mismatch negativity (Flip-Flop) procedure

After a recovery period of 2 weeks, a baseline EEG was recorded using the active two-electrode differential amplifier (Biosemi, Netherlands) for 20 min without acoustic stimuli. Auditory stimuli were generated with a custom program written in Labview and sound stimuli were delivered through a speaker mounted above the floor of the experimental box. For each box, sound-pressure level and frequencies were checked with a high-quality sound meter (Brüel & Kjar 2270-S, HBK) according to a standardized protocol. The EEG signals underlying the auditory-evoked potentials were digitized at 512 Hz, band-pass filtered between 0.16 Hz and 100 Hz. Vigilance states were identified using EEG amplitude and spectra records reconstituted on the computer screen. Sleep–wake states were determined in 2-s epochs using both visual evaluation of the recordings, and scattergram plots of EEG power. Histograms were sorted on the vigilance state at the stimulus onset and a waking state-specific average was generated across the recordings. Intervals of EEG recordings spanning time from 50 ms before to 450 ms after stimulus onset were extracted, baseline-corrected, and averaged ERP waveforms were computed for each animal and stimulus condition, while the average across animals resulted in grand average ERPs. Epochs with the EEG signal that exceeded a difference of 1000 μV within 200 ms in each segment were rejected. For deviance detection, pure 6- and 8-kHz tones of 20-ms duration, were delivered at about 80-dB sound-pressure level through a loudspeaker placed above the animal in the cage. A series of four randomized sequences of 20 min were presented at 3-s interstimulus intervals, with the low-pitch tone of 6 kHz serving as the frequent (STD, probability 80%) and the high-pitch tone of 8 kHz as the infrequent oddball (DEV, probability 20%), and vice versa as a means to control for sound frequency (i.e., STD is delivered as DEV: flip-flop design). This protocol was determined during a harmonization effort, based on data collected from experiments performed in multiple sites involved in the IMI PRISM consortium.

The peak amplitudes N1 or P2 components of the ERPs were extracted from 20 to 60 ms or 50 to 125 ms after stimulus presentation, for each mouse strain, and averaged across genotype groups.

### Statistical analysis

#### Human data

For human EEG data, four group pairs were created: SZ and AD, SZ and YC, AD and AC, and AC and YC. The Grubbs test was used to detect and reject outliers. Group comparisons of EEG average amplitude were performed for each pair at each electrode using a two-sample two-tail Student’s *t* test. The results of the statistical analyses are presented as brain heatmaps. For all analyses, a *P* value below 0.05 was considered significant.

#### Animal data

Rat data were analyzed by Student’s *t* test or two-way repeated measures ANOVA, depending on the experimental design. Where appropriate, post hoc analyses were carried out with a Sidak’s test. All reported *P* values are two-sided. The normality of the variables was assessed by Shapiro–Wilk test and the homoscedasticity was assessed by Levene’s test.

For each parameter measured, animals presenting individual values outside mean ± 2 SD were considered outliers and discarded from the statistical analysis of that parameter. Based on this criterion, two rats (both from the vehicle group) were discarded from NOR analyses, while three rats (two from the vehicle group, one from the PCP group) were discarded from the MMN analyses.

For the mouse data, the peak amplitudes of N1 or P2 components of the ERPs were extracted from 20 to 60 ms and 100 to 150 ms, respectively. The MMN area under the curve was derived at 50–125 ms after stimulus presentation, for each mouse strain, and averaged across genotype groups. Mean peak values of N1 or P2 and area under curves were analyzed using one-way ANOVA followed by Bonferroni’s posttest (when appropriate).

## Results

### ERP profiles in SZ and AD patients

**Fig. 2:**
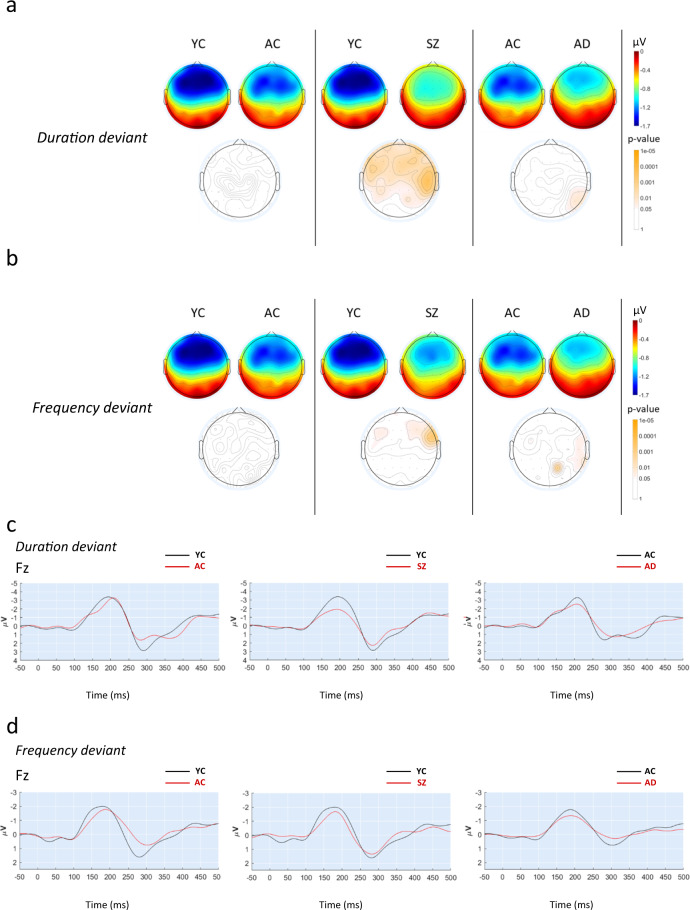
MMN response in healthy controls, SZ, and AD patients. Brain topographic maps for all electrodes showing MMN average amplitude during the auditory oddball paradigm (50-ms standard tones of 1000 kHz, 100-ms duration-deviant tones of 1000 kHz, and 50-ms frequency-deviant tones of 1050 Hz) in young controls (YC), aged controls (AC), Schizophrenia patients (SZ), and Alzheimer’s disease patients (AD) for duration deviants (**a**) and frequency deviants (**b**). Below each pannel, the corresponding p-values are shown as brain heatmaps and obtained for the indicated comparisons using a two-sample two-tail Student’s *t* test. The results are expressed as means. (**c**) Grand average-evoked potentials elicited during the auditory oddball stimulation and illustrating MMN average amplitude at the Fz electrode for duration and frequency deviants (**d**) in young control (YC), aged control (AC), schizophrenic patients (SZ), and Alzheimer’s patients (AD). The results are expressed as mean and negativity is plotted as an upward deflection.

For clarity purpose, only data of MMN average amplitude are described in this study and are shown in Fig. 2 (data not shown for peak amplitude, peak latency, and AUC).

Figure 2a, b shows that, despite a slight reduction of MMN responses in the frontoparietal area of aged control (AC) subjects for both types of deviants, no significant difference was observed between young control (YC) and AC (e.g., at the Fz electrode: −2.57 ± 1.47 µV and −1.56 ± 1.00 µV in YC for the duration and frequency deviants, respectively vs. −2.33 ± 1.39 µV and −1.36 ± 1.07 µV in AC). As expected, comparison of SZ patients with age-matched control (YC) revealed a significant attenuation of MMN response, especially in the front-parietal areas for duration deviants (e.g., −1.59 ± 1.14 µV in SZ vs. −2.57 ± 1.47 µV in YC at the Fz electrode) as shown in Fig. 2a. With frequency deviants, this effect was much lower and hardly statistically significant in a limited temporal unilateral area (e.g., −1.10 ± 0.88 µV in SZ vs. −1.56 ± 1.00 µV in YC at the Fz electrode) as shown in Fig. 2b. No significant difference was observed when comparing AD patients with age-matched controls (AC) (e.g., at the Fz electrode: −2.17 ± 1.20 µV and −0.92 ± 0.70 µV in AD for the duration and frequency deviants, respectively vs. −2.33 ± 1.39 µV and −1.36 ± 1.07 µV in AC), except for two scarce parietal and occipital areas where a significant increase in MMN average amplitude was observed (Fig. 2a, b).

Figure 2c, d illustrates the morphology of the ERP obtained from Fz electrode. All groups exhibited a negative potential to deviants around 200-ms latency that was reduced in SZ patients compared to YC with duration deviant (Fig. 2c) but less clearly with frequency deviant (Fig. 2d). A positive wave peaking slightly before 300 ms was also observed in all groups and seemed to be impaired in AC compared to YC (Fig. 2c, d).

### Back-translation into rodent models

**Fig. 3:**
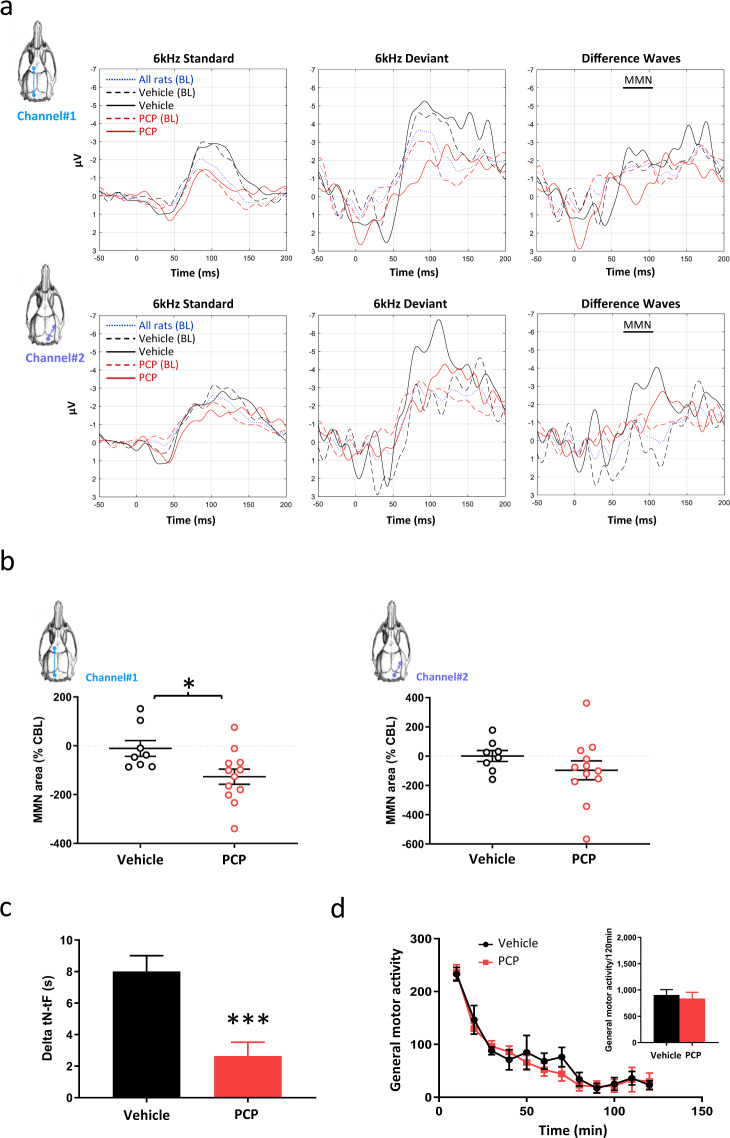
MMN response in rats after PCP withdrawal. **a** Grand average-evoked potentials elicited during auditory passive oddball stimulation flip-flop protocol. Changes on standards and deviants’ stimuli (standard tones of 8 kHz presented at 90% probability; deviant tones of 6 kHz presented at 10% probability) at baseline (i.e., prior subchronic treatment) in all animals (*n* = 23, blue dot line), animals from the PCP (*n* = 13, red dash line), or vehicle (*n* = 10, black continuous line) and 7 days after PCP (*n* = 12, red continuous line) or vehicle (*n* = 8, black continuous line) treatment discontinuation. The upper and lower panels illustrate ERPs recorded via Channel#1 and Channel#2, respectively. The results are expressed as means. BL baseline. **b** The left panel illustrates MMN area (calculated with the 6-kHz flip-flop method) expressed in change from baseline (CBL) with EEG recording on Channel#1 (*n* = 8–12). An unpaired Student’s *t* test revealed significant impairment in PCP-treated rats compared to control (*t*_18_ = 2.496; *P = 0.0225). Individual data are represented along with means ± SEM. The right panel shows MMN area expressed in change from baseline (CBL) with EEG recording on Channel#2 (*n* = 8–12). An unpaired Student’s *t* test revealed no significant impairment in PCP-treated rats compared to control (*t*_18_ = 1.145; *P* = 0.2671). Individual data are represented along with means ± SEM. **c** Delta tN–tF in the NOR assay (*n* = 11–14). An unpaired Student’s *t* test revealed significant impairment in PCP-treated rats compared to control (*t*_23_ = 4.007; ***P = 0.0006). The results are expressed as means ± SEM. **d** General locomotor activity (number of photobeam breaks, *n* = 11–14). No significant effect of the subchronic PCP treatment was observed in the 10-min binning (*F*_1,25_ = 0.1734; *P* = 0.681 for treatment effect with two-way ANOVA) or the 120-min (*t*_25_ = 0.416; *P* = 0.681 with unpaired Student t-test) analyses. The results are expressed as means ± SEM.

Figure 3a illustrates the response evoked by the 6-kHz tones. The morphology of the ERPs obtained at baseline (i.e., prior treatment) was similar in both experimental groups (black and red dash lines) and stable within the vehicle group when comparing baseline (black dash line) to post treatment (continuous black line). The blue dash line shows the grand average auditory-evoked potentials obtained at baseline (i.e., prior to any subchronic treatment) in all rats during the oddball/flip-flop stimulation protocol with both recording channels. In both channels (though in a clearer extent on Channel#1), the MMN amplitude shows a negative component around 80 ms post stimulus, which is also observed (but around 200 ms) in healthy human subjects (Fig. 2d). This ERP morphology can be also seen after vehicle subchronic treatment (continuous black line), while animals exposed to PCP (continuous red line) exhibited attenuated MMN similarly to SZ patients (Fig. 2b).

Figure 3b reporting baseline-normalized MMN area (i.e., individual MMN area post treatment against MMN area prior treatment in the same individual) obtained with the 6-kHz deviant (descending oddball) indicates that subchronic PCP treatment was associated with a significant MMN attenuation when compared to rats subchronically treated with the vehicle when EEG was recorded via Channel#1 (−11.0% change from baseline in vehicle- vs. −126.7% in PCP-treated rats; *t*_18_ = 2.496; *P* = 0.0225). This effect was much more modest and not significant when MMN response was recorded via Channel#2 (−5.9% change from baseline in vehicle- vs. −96.6% in PCP-treated rats; *t*_18_ = 1.145; *P* = 0.2671). Interestingly, analyses of the response to the 8-kHz deviant (ascending oddball) revealed higher variability with no robust difference between vehicle- and PCP-treated animals (Supplementary Data).

It should be noted that three rats (one from the PCP group, two from the vehicle group) exhibited post-treatment MMN area as well as change from baseline MMN area outside mean ± 2 SD in their group for both descending and ascending oddballs and for both recording channels. These animals were considered as outliers (see “Statistical analysis” section) and discarded from analyses described above.

Another cohort of rats was used to control the behavioral effect of the PCP treatment scheme being used in the EEG evaluation. Figure 3c illustrates a significant deficit in the NOR assay (delta was 8.0 s in vehicle rats vs. 2.64 s in PCP rats; *t*_23_ = 4.007; *P* = 0.0006), which could not be due to any motor impairment (Fig. 3d) after subchronic PCP treatment.

**Fig. 4:**
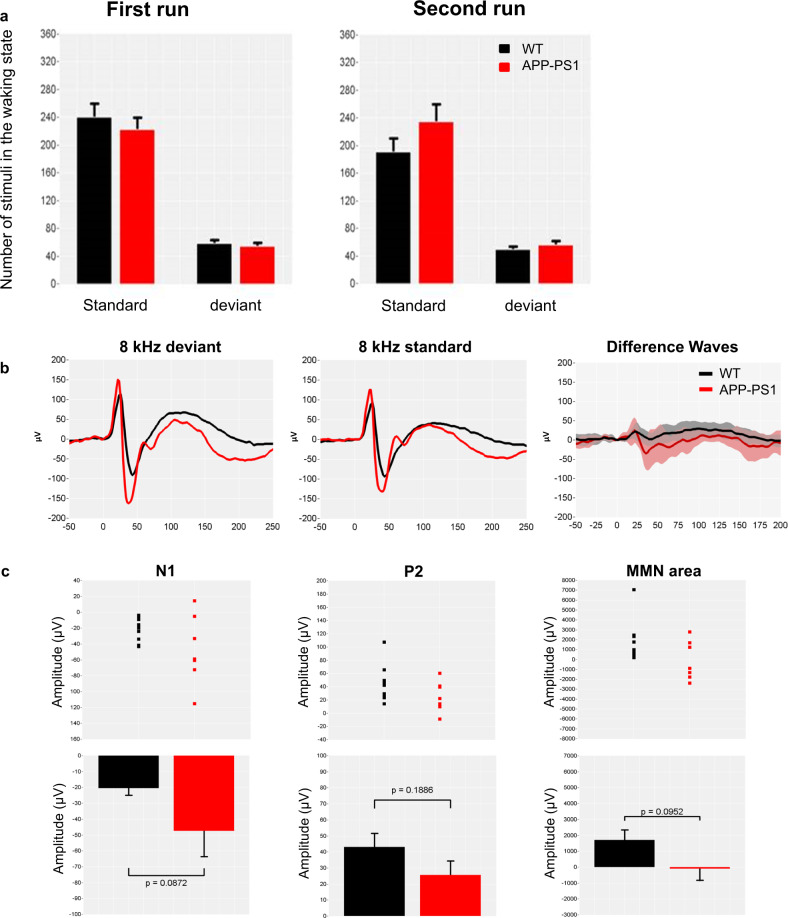
MMN response in APP/PS1 mice. Grand average-evoked potentials elicited during auditory passive oddball stimulation flip-flop protocol in 13-month-old APP/PS1 and wild-type (WT) mice. **a** Number of trials categorized as wakefulness for both runs are shown, whereas the remaining AEP responses were discarded from the final analysis. **b** Changes on standards and deviants’ stimuli (standard tones of 8 kHz presented at 80% probability; deviant tones of 8 kHz presented at 20% probability) in both genotypes; top-left and middle plots, on difference N1 and P2 waveforms at 20–60 ms and 100–150 ms after stimulus presentation, respectively; top right, on the MMN area at 50–125 ms post stimuli presentation. Average-evoked auditory responses had similar waveforms during the waking state. **c** Scatterplots of the individual N1 and P1 responses and for the AUC of the N1 differential responses (middle plots). Bar charts showing the mean amplitude values for N1 and P1 responses and for the AUC of the N1 differential responses (bottom plots). Data from three APP/PS1 mice were excluded from the group analysis: in one animal, one electrode was defected and produced artifacts, whereas AEP components were not detectable in two animals. Mean amplitude data are presented as mean + SEM.

Figure 4 recapitulates the data collected in APP/PS1-21 mice. Vigilance states were scored in 2-s epochs on the basis of the EEG, and each trial of the sound presentation was categorized as occurring during either wakefulness or sleep states. The mean number of standard and deviant stimuli occurring during waking states is presented in Fig. 4a. There were no major differences in the number of trials categorized as wakefulness in both runs for WT and APP/PS1 mice (run 1: standard: 240.1 ± 19.7 vs. 222.7 ± 16.5; deviant: 58.1 ± 4.8 vs. 54.0 ± 4.7, run 2: 191.1 ± 19.1 vs. 234.8 ± 24.6; deviant: 49.1 ± 4.6 vs. 55.4 ± 6.2, respectively). In line with previous data^32,33^, the grand average auditory-evoked potentials (AEP) show an early positive peak component (P1) at about 25 ms post stimulus, followed by a prominent negative peak component (N1) at 45 ms, a positive peak (P2) at about 145 ms (Fig. 4b). AEP responses to the first standard after the deviant (non- or less-adapted response), which might be regarded as a control and the last standard before the next deviant (fully adapted response), were extracted and were compared to responses elicited by all standard stimuli. The amplitude of the ERPs to standard stimuli did not reveal the qualitative difference, suggesting that no habituation to the continuously displayed tones was present over the time of the experiment (data not shown). An MMN-like potential response peaking at 50–125 ms was observed in both genotypes for the high deviant tone versus the low standard tone (Fig. 4b) but not for the reversed contrast (not shown). We performed an iterative approach to determine the most significant difference between the AUC (WT vs. APP-PS1 mice) in different time windows and to determine the most interesting time window for analysis. The MMN AUC selected fits within the time interval that was reported in other animal studies, where mismatch responses were characterized as negative deflections^34^. No difference was found for peak amplitudes of N1 (*F*_1, 15_ = 3.3, *P* = 0.0872) and P2 components (*F*_1,15_ = 1.8, *P* = 0.1886), as well as for MMN area (*F*_1,15_ = 3.1, *P* = 0.0952) (Fig. 4c).

## Discussion

In this study, we used a circuit-based transnosographic approach providing new insight into the cross-disease evaluation of the MMN response. We report clinical data that we back-translated into animal disease models. Our study strongly supports the translational potential of MMN and emphasizes its tremendous interest as efficacy biomarker for neuropsychiatry drug development. Although there is a plethora of studies investigating MMN response in SZ (for review see refs. ^12,13,35–37^) and associated animal models^28,35,38,39^ as well as in AD patients (for review see refs. ^12,13,36^), a more limited amount of MMN data is available for AD animal models^32^. While we confirm the sensitivity of MMN observation in schizophrenic patients and preclinical PCP models, the present study discloses novel MMN results on the IMI PRISM dataset and the APP/PS1-21 mouse model of amyloidosis.

The MMN paradigm has gained considerable interest in cognitive neuroscience as it provides an experimental window to the mechanisms underlying sensory information processing via deviance detection^40^. The knowledge about regions involved in its generation, its simplicity, and sensitivity leads to the widespread use of MMN clinical research when investigating patients with communication or attention problems. Deficits in MMN response have been associated with schizophrenia as well as with numerous brain disorders^15,41^ (for review see refs. ^12,13,36^). Interestingly, MMN-like response has been successfully demonstrated in nonhuman primates and rodents^42^, while attempts have been made to develop animal models of deficits in MMN response. Rodent models have been developed such as those involving a direct pharmacological manipulation to induce N-methyl-D-aspartate (NMDA) receptor hypofunction^28,43–45^ and models involving abnormal neurodevelopment^39^ as well as genetic alteration of the NMDAr signaling in mice^46,47^.

### Schizophrenia

Consistently with the literature^15,41,48^, our data demonstrate that MMN was impaired in SZ patients compared to age-matched healthy controls. It should be noted that this impairment was much clearer with a duration deviant than with a frequency deviant. This finding is consistent with a line of evidence suggesting that MMN reduction due to duration deviant is more severe than with pitch deviant, especially at earlier stages of the disease^49–52^.

Importantly, we were able to back-translate the MMN deficit observed in SZ patients into a PCP- withdrawal rat model of SZ previously described^29,53,54^. It is worth noting here that, in this model, a preliminary study revealed that frequency but not duration deviants allowed eliciting a MMN-like response (data not shown). Our data demonstrate that, similarly to healthy subjects from the clinical trial, rats tested prior to PCP exposure and/or after vehicle treatment exhibited a brain response to deviant MMN-eliciting stimuli that was differentiated from the response to repetitive standards. Although the amplitude of the MMN response was in the same range for both species (i.e., around −4 µV), the latencies of the different components slightly differed as shown by the early positive wave around 40 ms observed in the rat but not in human and a negative component around 90 ms also observed by others and thought to reflect MMN in the rat^27,28^, while the MMN peak was generally observed around 200 ms in human. More interestingly, the MMN deficit observed in SZ patients (when compared to healthy young control) was equally observed in rats after cessation of subchronically PCP treatment (when compared to vehicle-treated rats). This effect of PCP on MMN is also in line with previous data reporting impaired MMN in rats under chronic PCP treatment^28^ or repeatedly exposed to PCP only during neonatal period^38^. Interestingly, the effect of PCP withdrawal on the MMN response was much clearer when EEG was recorded with the active electrode onto the frontal cortex (Channel#1), whereas variability was much higher when the recording was made from a more posterior area (i.e., via Channel#2 that corresponded to the active electrode onto the auditory cortex). This finding is consistent with the data obtained in human studies suggesting a more clear difference in the frontal area, while occipital areas seemed to be less impacted (Fig. 2a, b).

Subchronic PCP treatment has been widely used to model schizophrenia in rodents as it is thought to some extent to resemble the symptomatology and neuropathology of the disease^29,53,54^. Notably, previous work reported decreased NMDAr binding when a wash-out period is implemented after subchronic PCP treatment^55^, which is consistent with the NMDAr hypofunction suggested by the MMN attenuation observed 7 days after PCP treatment discontinuation in the present study. Furthermore, different papers report that such PCP regime as well as similar treatment scheme with other NMDAr antagonists were associated with a decreased number of hippocampal (as well as cortical) parvalbumin (PV)-positive neurons (for review see refs. ^53,54,56^). These fast-spiking GABAergic interneurons are thought to be particularly sensitive to NMDAr antagonists, notably because of the cortical excitation observed after acute NMDAr antagonist administration, which is possibly due to a preferential reduction in their firing, resulting in the disinhibition of excitatory pyramidal neurons^56–58^. A link between inhibition of GABAergic tone mediated by NMDAr hypofunction on interneurons and glutamatergic hyperexcitation has been proposed in a rat model of NMDAr antagonist repeated exposure^59^, and it has been reported that cortical PV- (along with somatostatin-) positive neurons influence representations of auditory stimuli (see Ross and Hamm for review^60^). Therefore, the NMDAr hypofunction suggested by the MMN attenuation observed in this study might reflect a probable PCP-induced alteration of PV-positive neurons, which is thought to resemble an aspect of SZ^56^.

Interestingly, in addition to the effects on MMN response, PCP withdrawal, was also associated with a working memory deficit. This finding is in line with data reviewed elsewhere^53^ and supports the idea that the MMN response is associated with cognitive performance^13,36,37^.

### Alzheimer’s disease

Inconsistent MMN results have been observed in AD. Some studies have reported no significant differences between controls and AD patients^61^, while others demonstrate a significant decline in sensory-memory trace in AD using long interstimulus intervals^62^, and propose MMN as a sensitive prognosis marker predicting the conversion of mild cognitive impairment to AD^63–66^. In our study, we did not observe MMN attenuation in AD patients when compared to their age-matched controls. Multiple factors may explain such contrast with aforementioned studies such as the nature of the auditory MMN procedure (especially the length of the interstimulus interval) and/or the characteristics of the population. For instance, Lindín and coworkers^63^ have reported a decreased MMN amplitude in amnestic mild cognitive impairment patients only in their middle-aged subgroup (50–64 years) but not in an older cohort (≥65 years old). The authors observed an age-related decrease in MMN amplitude also found by others^67^ and suggest that this may be due to an age-related decline in the mechanism for echoic memory trace maintenance and/or the preattentional mechanisms. This may explain the lack of AD-related MMN impairment observed in the present study (as suggested also by the slight, but not significant, decreased MMN amplitude observed in aged control compared to young). Furthermore, while the 600-ms interstimulus interval being used in this study corresponds to standard protocols utilized in SZ patients, it might have been too short to allow seeing MMN impairment in AD patients. Such assumption is notably supported by the report from Pekkonen and coworkers, which describes the importance of having long (≥3 s) rather than shorter (<1 s) interstimulus intervals to observe AD-related MMN attenuation^62^.

It is noteworthy mentioning that we also have observed such mixed results in animals when comparing our earlier^32^ and the present studies. The contrast between these two data sets might be related to animal models and pathology studied as well as to the experimental design. Accordingly, in the earlier work, a model of tauopathy using preformed fibrils seeded in the hippocampus showed deficits in the peak N amplitude of the MMN response at an age with blown tauopathy^32,68^, while in this study, an animal model of amyloid overexpression, recorded at the age of post-plaque deposits, did not exhibit deficits in MMN response. Concerning the experimental design, in the fibril seeding study, we have used a standard passive 2-tone auditory passive oddball protocol with quasi-random sequence of frequent and infrequent target tones^32^, while in the present work, we have used the traditional way to evoke MMN with a frequency-mismatch “flip-flop” design in which the frequencies of standard and deviant acoustic stimuli are swapped in two consecutive sessions^27^: a protocol that is in line with the protocol being used in the rat experiment (and somehow with the PRISM clinical trial that uses similar protocol in AD and SZ patients). One hypothesis explains the lack of alterations in MMN response in APP/PS1 animals by the age of the animals at which the recording took place. On the one hand, APP/PS1 mice developed selective deficits of spatial memory at 8 months of age^69,70^, which became worse at the age of 22 months. A significant impairment in glucose metabolism detected in the hippocampus of 22-month-old APP/PS1 mice corresponded well to the spatial memory deficit. However, no significant metabolic deficit was found in the motor or sensory cortical areas in APP/PS1 mice and their performance on locomotor testing did not differ from the performance of age-matched WT controls^71^, suggesting that this measure may be relatively insensitive at detecting subtle changes in nonspatial cognitive component. It would be also of interest to control whether aging per se can influence MMN response in healthy rodents as we could not find any published study addressing this question. On the other hand, an anomalous MMN response has been associated with glutamatergic abnormalities and NMDAr hypofunction^72^. Earlier stages of AD are characterized by an upregulation of synaptic components contributing to increased glutamate signaling. The APP/PS1 AD mouse model showed increased stimulus-evoked glutamate release at younger ages, which then steadily decreases with age and Aβ accumulation^73,74^. Later stages of AD show markedly decreased glutamatergic activity, which is in sharp contrast to the earlier period of glutamatergic hyperactivity; therefore, it is conceivable that the MMN response observed in the present study correlates with the age range. For interpretation of the results obtained with the APP/PS1 model used in this study, similarly to other AD pathology and symptomatic animal models (e.g., APP overexpression, APP knock-in, and tau aggregation, cholinergic), the nature of the pathology, as well as the stage of pathology progression, should be considered a crucial driver for obtaining functional/symptomatic effects and hence add to the complexity of adequate back-translation of clinical findings.

All studies in the present paper were conducted within the framework of the European project IMI PRISM, which attempts to identify the underlying neurobiology of neuropsychiatric symptoms, especially social withdrawal, associated with SZ and AD utilizing a novel transdiagnostic approach^8,11,12^. The overall goal is to further understand the physiopathology of these disorders and possibly identify subpopulations where personalized medicine approaches may be applied. Our results highlight MMN attenuation in SZ but not in AD patients, whereas previous reports suggest that MMN response could be associated with cognitive performance across multiple CNS disorders^13,36,37^. This contrast is likely due to the nature of the auditory MMN procedure utilized in our study, and especially the 600-ms interstimulus interval, which was standard with respect to SZ patients but possibly too short for AD patients (see above). It would be of interest to evaluate whether an auditory MMN procedure with a longer interstimulus interval could allow observing MMN attenuation both in SZ and AD patients. Another appealing track relies on the evaluation of ASSR, which is also known to be altered in SZ and AD patients (for review see ref. ^12^) and can be measured in animal models^75–79^.

One of the major challenges in neuropsychiatry drug development is the poor translation of nonclinical findings to clinical outcomes^1–3^. The present work aligns with the need to further develop back-translational approaches focusing on evolutionarily preserved neurobiological/neuropharmacological systems that may help addressing this challenge^11^. Our data do not support the identification of cross-disorder neurobehavioral subtypes that could help patients’ clustering based on a biological rational rather than pure symptom observation for precision medicine intervention. However, this work suggests further investigations (e.g., adaptation of MMN protocol, evaluation of another EEG biomarker such as ASSR) that may allow addressing this challenge. Furthermore and importantly, here we provide evidences that MMN could be used as a quantitative/objective efficacy readout during both preclinical and clinical stages of SZ drug development. This strongly emphasizes the translational value of MMN and assessing alterations in deviance detection as a biomarker for CNS drug development in the current context of a legitimate debate around the validity of animal models available (and/or the way they have been used so far)^1,3,8,9,11,80^.

## Supplementary information

MMN response in rats after PCP withdrawal (8 kHz flip flop)
