# Correction: Mismatch negativity as EEG biomarker supporting CNS drug development: a transnosographic and translational study

**DOI:** 10.1038/s41398-021-01408-5

**Published:** 2021-05-10

**Authors:** Simon Loiodice, Wilhelmus H. Drinkenburg, Abdallah Ahnaou, Andrew McCarthy, Geoffrey Viardot, Emilie Cayre, Bertrand Rion, Valérie Bertaina-Anglade, Marsel Mano, Philippe L’Hostis, Christophe Drieu La Rochelle, Martien J. Kas, Philippe Danjou

**Affiliations:** 1 Biotrial Pharmacology, 7-9 rue Jean-Louis Bertrand, 35042 Rennes, France; 2 Department of Neuroscience Discovery, Janssen Research & Development, a Division of Janssen Pharmaceutical NV, Turnhoutseweg 30, B-2340 Beerse, Belgium; 3 Groningen Institute for Evolutionary Life Sciences, University of Groningen, P.O. Box 11103, 9700 CC Groningen, The Netherlands; 4 Lilly Research Laboratories, Windlesham, Surrey GU20 6PH UK; 5 Biotrial Neuroscience, Avenue de Bruxelles, 68350 Didenheim, France

**Keywords:** Neuroscience, Biomarkers

Correction to: *Translational Psychiatry*

10.1038/s41398-021-01371-1 published online 29 April 2021

The original version of this article unfortunately contained a mistake in an author name. The correct name is Abdallah Ahnaou. The original article has been corrected.

