# Supplementary material for: Mismatch negativity as EEG biomarker supporting CNS drug development: a transnosographic and translational study
Source: Transl Psychiatry. 2021 Apr 29;11:253. doi: 10.1038/s41398-021-01371-1 (PMC8085207; doi:10.1038/s41398-021-01371-1)
Supplement: Supplementary file 1 — MMN response in rats after PCP withdrawal (8 kHz flip flop) [file 41398_2021_1371_MOESM1_ESM.pptx]

## Slide 1
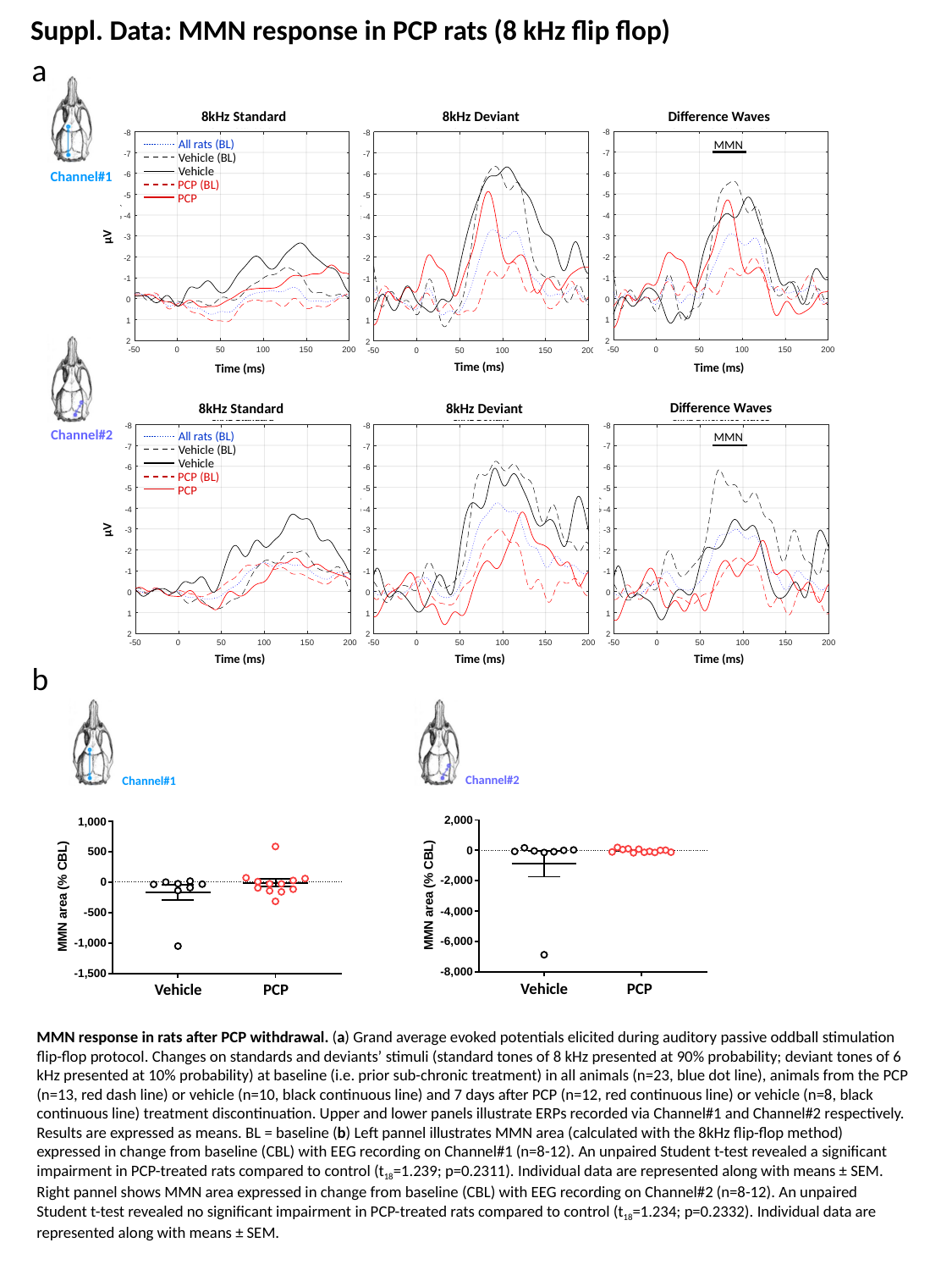

Suppl. Data: MMN response in PCP rats (8 kHz flip flop)
a
Difference Waves
8kHz Deviant
8kHz Standard
All rats (BL)
Vehicle (BL)
Vehicle
PCP (BL)
PCP
MMN
Channel#1
µV
Time (ms)
Time (ms)
Time (ms)
Difference Waves
8kHz Deviant
8kHz Standard
Channel#2
All rats (BL)
Vehicle (BL)
Vehicle
PCP (BL)
PCP
MMN
µV
Time (ms)
Time (ms)
Time (ms)
b
Channel#2
Channel#1
Vehicle
PCP
Vehicle
PCP
MMN response in rats after PCP withdrawal. (a) Grand average evoked potentials elicited during auditory passive oddball stimulation flip-flop protocol. Changes on standards and deviants’ stimuli (standard tones of 8 kHz presented at 90% probability; deviant tones of 6 kHz presented at 10% probability) at baseline (i.e. prior sub-chronic treatment) in all animals (n=23, blue dot line), animals from the PCP (n=13, red dash line) or vehicle (n=10, black continuous line) and 7 days after PCP (n=12, red continuous line) or vehicle (n=8, black continuous line) treatment discontinuation. Upper and lower panels illustrate ERPs recorded via Channel#1 and Channel#2 respectively. Results are expressed as means. BL = baseline (b) Left pannel illustrates MMN area (calculated with the 8kHz flip-flop method) expressed in change from baseline (CBL) with EEG recording on Channel#1 (n=8-12). An unpaired Student t-test revealed a significant impairment in PCP-treated rats compared to control (t18=1.239; p=0.2311). Individual data are represented along with means ± SEM. Right pannel shows MMN area expressed in change from baseline (CBL) with EEG recording on Channel#2 (n=8-12). An unpaired Student t-test revealed no significant impairment in PCP-treated rats compared to control (t18=1.234; p=0.2332). Individual data are represented along with means ± SEM.
